# Non-Invasive Estimation of Glioma *IDH1* Mutation and *VEGF* Expression by Histogram Analysis of Dynamic Contrast-Enhanced MRI

**DOI:** 10.3389/fonc.2020.593102

**Published:** 2020-12-08

**Authors:** Yue Hu, Yue Chen, Jie Wang, Jin Juan Kang, Dan Dan Shen, Zhong Zheng Jia

**Affiliations:** Department of Medical Imaging, Affiliated Hospital of Nantong University, Nantong, China

**Keywords:** glioma, isocitrate dehydrogenase, vascular endothelial growth factor, magnetic resonance imaging, histogram

## Abstract

**Objectives:**

To investigate whether glioma isocitrate dehydrogenase (*IDH*) 1 mutation and vascular endothelial growth factor (*VEGF*) expression can be estimated by histogram analysis of dynamic contrast-enhanced magnetic resonance imaging (DCE-MRI).

**Methods:**

Chinese Glioma Genome Atlas (CGGA) database was wined for differential expression of *VEGF* in gliomas with different *IDH* genotypes. The *VEGF* expression and *IDH1* genotypes of 56 glioma samples in our hospital were assessed by immunohistochemistry. Preoperative DCE-MRI data of glioma samples were reviewed. Regions of interest (ROIs) covering tumor parenchyma were delineated. Histogram parameters of volume transfer constant (*K^trans^*) and volume of extravascular extracellular space per unit volume of tissue (*V_e_*) derived from DCE-MRI were obtained. Histogram parameters of *K^trans^*, *V_e_* and *VEGF* expression of *IDH1* mutant type (*IDH1^mut^*) gliomas were compared with the *IDH1* wildtype (*IDH1^wt^*) gliomas. Receiver operating characteristic (ROC) curve analysis was performed to differentiate *IDH1^mut^* from *IDH1^wt^* gliomas. The correlation coefficients were determined between histogram parameters of *K^trans^*, *V_e_* and *VEGF* expression in gliomas.

**Results:**

In CGGA database, *VEGF* expression in *IDH^mut^* gliomas was lower as compared to wildtype counterpart. The immunohistochemistry of glioma samples in our hospital also confirmed the results. Comparisons demonstrated statistically significant differences in histogram parameters of *K^trans^*and *V_e_* [mean, standard deviation (SD), 50th, 75th, 90th. and 95th percentile] between *IDH1^mut^*and *IDH1^wt^*gliomas (*P* < 0.05, respectively). ROC curve analysis revealed that 50th percentile of *K^trans^* (0.019 min^−1^) and *V_e_* (0.039) provided the perfect combination of sensitivity and specificity in differentiating gliomas with *IDH1^mut^*from *IDH1^wt^*. Irrespective of *IDH1* mutation, histogram parameters of *K^trans^*and *V_e_* were correlated with *VEGF* expression in gliomas (*P* < 0.05, respectively).

**Conclusions:**

*VEGF* expression is significantly lower in *IDH1^mut^* gliomas as compared to the wildtype counterpart, and it is non-invasively predictable with histogram analysis of DCE-MRI.

## Introduction

Glioma is the most common primary intracranial tumor. According to the 2016 World Health Organization (WHO) classification criteria, mutation in the gene encoding isocitrate dehydrogenase (*IDH*) enzyme has been identified in the sub-stratification of glioma (1). Emerging evidence has shown that *IDH* mutation can convert *α*-ketoglutarate (*α*-KG) to 2-hydroxyglutaate (2-HG). Excessive 2-HG can activate prolyl hydroxylase (PHD) and then promote the degradation of hypoxia induced factor (HIF), which can weaken tumor microvascular proliferation (2, 3). Moreover, a study has reported that *IDH* mutation inhibited PI3K/Akt signaling and reduced the HIF expression level (4). Vascular endothelial growth factor (*VEGF*) regulated by HIF is one of the angiogenesis-related genes, and its over-expression correlates with poor prognoses in gliomas (5, 6). So, anti-*VEGF* therapy increases progression-free survival and improves quality of life in patients with glioma (7, 8). On account of these findings, we assumed that the expression level of *VEGF* was lower in *IDH* mutant type (*IDH^mut^)* gliomas, which fitted with the indolent clinical course of *IDH^mut^*gliomas. *IDH* genotypes and the expression level of *VEGF* can be detected by surgery or biopsy, but it suffers from several drawbacks such as sampling error, tumor heterogeneities, and risk of surgical complications. Therefore, it is urgent to find a non-invasive technique. The pharmacokinetic parameters derived from dynamic contrast-enhanced magnetic resonance imaging (DCE-MRI) have the ability to predict the microvascular features of glioma in a non-invasive manner (9, 10). Based on the value of DCE-MRI in gliomas and the hypothetical correlation between glioma *IDH* mutation and *VEGF* expression, we aimed to investigate whether glioma *IDH1* mutation and *VEGF* expression can be estimated by histogram analysis of DCE-MRI.

## Materials and Methods

### Chinese Glioma Genome Atlas Database Analysis

The data of *VEGF* expression and *IDH* genotypes in 641 unique samples with glioma (286 samples with *IDH^mut^* and 355 with *IDH* wildtype (*IDH^wt^*)) were available from the CGGA database. Wilcox test implemented in R language was utilized to analyze the differential expression of *VEGF* in gliomas with different *IDH* genotypes.

### Glioma Samples

The inclusion criteria for case selection of this study were as follows: (1) pathologically confirmed gliomas; (2) treatment-naive before MRI examination and surgery; (3) underwent preoperative DCE-MRI. The final study population included 56 patients. All the data were retrospectively reviewed. The clinical cohort consisted of 33 males and 23 females, aged 55 ± 14 years (range, 22–75 years). This retrospective data evaluation was approved by the local institutional review board and written informed consent was obtained from each patient.

### MRI

MRI was examined on a 3.0-T MR system (GE Healthcare, Milwaukee, WI, USA) with a 16-element head-neck coil. Conventional MRI were T1-weighted imaging, T2-weighted imaging, T2-weighted fluid attenuated inversion recovery (T2-Flair) imaging.

DCE-MRI was done using dynamic scan of a T1-fast field echo (T1-FFE; RF-spoiled gradient echo) sequence and setting the following parameters: repetition time (TR), 5.1 ms; echo time (TE), 1.4 ms; slice thickness, 2.8 mm; matrix, 256 × 210; field of view (FOV), 250 mm × 250 mm; axial scanning. Precontrast images with multiple flip angles 3, 6, 9, 12, and 15° were acquired for the T1 maps. Then, the contrast agent (Ommiscan, GE Healthcare, Oslo, Norway) was administered (0.1 mmol/kg of body weight) through the antecubital vein *via* a power injector at 4 ml/s, followed by a flush of 15 ml saline. A series of 1,000 images at 50 time points for 20 axial sections were acquired with a temporal resolution approximately of 7 s for each time point. Finally, the postcontrast T1-weighted imaging was conducted in the same axial geometry.

### Image Analysis

A software module (OmniKinetics, GE Healthcare, China) was applied for post-processing. The volume transfer constant (*K^trans^*) and volume of extravascular extracellular space per unit volume of tissue (*V_e_*) were obtained based on the extended Tofts and Kermode pharmacokinetic model. The arterial input function was located on the superior sagittal sinus. The postcontrast T1-weighted imaging functioned as reference. When the tumor was without enhancement, the T2-Flair images were registered in the DCE images, and the registration images were taken as reference. Regions of interest (ROIs) covering tumor parenchyma were placed after consensus was reached between two experienced radiologists. The ROIs placed on the reference images can automatically transfer onto the parameters maps, and then histogram parameters of *K^trans^* and *V_e_* were generated automatically.

### Pathology

All clinical glioma specimens were fixed in 4% formalin and embedded in paraffin. The paraffin blocks were cut into 5-μm sections and stained with hematoxylin and eosin (H&E).

The *IDH1* mutation was detected with a mutation-specific antibody. Staining was interpreted as positive when ≥10% of the tumor cells exhibited an intense cytoplasmic staining, whereas staining was deduced as negative when positive cells <10% (11).

Tissue blocks from each specimen were stained for VEGF rabbit anti-human polyclonal antibody (Beijing Bioss Biotechnology Co., Ltd.). All specimens were analyzed with Motic Images Advanced software (version 3.2, Motic China Group Co., Ltd). A semiquantitative grading of *VEGF* expression was applied. The percentage of positive cells was calculated in five hotspots randomly selected from each section, and the percentage was scored: 1) zero point: positive cells <5%; 2) one point: positive cells 5–25%; 3) two points: positive cells 26–50%; 4) three points: positive cells 51–75%; 5) four points: positive cells 76–100%. The staining intensity was also scored: 1) zero point: no staining; 2) one point: faint yellow; 3) two points: brown-yellow; 4) three points: brown. Total score = percentage score × staining intensity score: 1) zero point: negative (−); 2) one to four points: weak positive(+); 3) five to eight points: positive (++); 4) 9–12 points: strong positive (+++).

### Statistical Analysis

Statistical computations were carried out with the SPSS 22.0 (SPSS Inc, Chicago, IL, USA). Histogram parameters (mean, standard deviation (SD), 50th, 75th, 90^th^, and 95th percentile) of *K^trans^*, *V_e_* and *VEGF* expression of *IDH1^mut^* gliomas were compared with the *IDH1^wt^* gliomas using the Mann–Whitney U test. Receiver operating characteristic (ROC) curve analysis was plotted to differentiate *IDH1^mut^* from *IDH1^wt^* gliomas. The Spearman test was performed to calculate correlation coefficients between histogram parameters of *K^trans^*, *V_e_* and VEGF expression in gliomas. *P* < 0.05 was considered statistically significant.

## Results

### Differential Expression of *VEGF* in Gliomas With Different *IDH* Genotypes in CGGA Database

**Figure 1:**
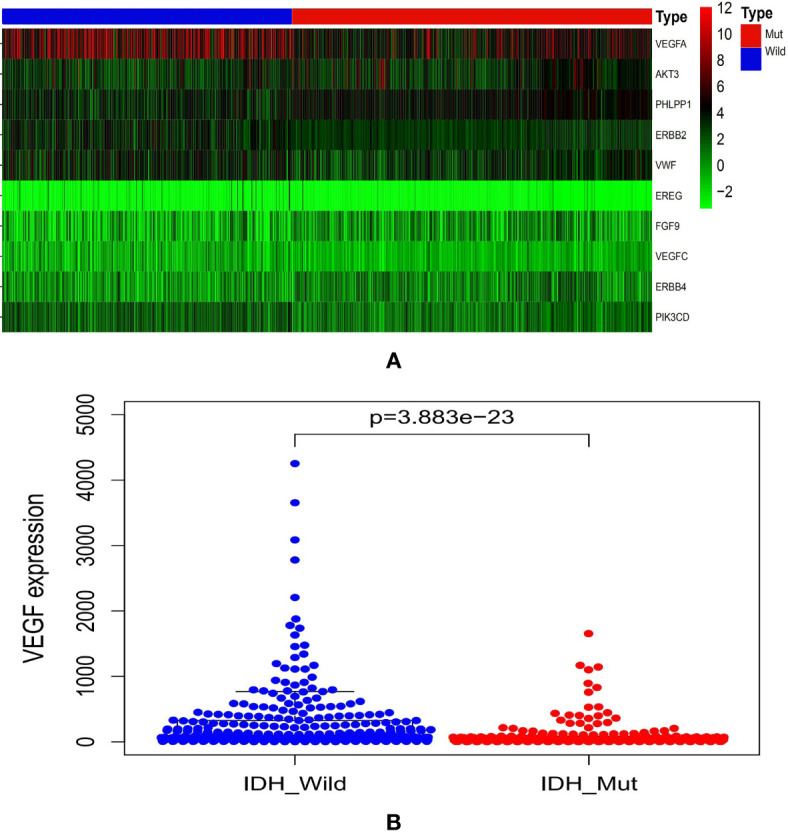
Graphs showed the differential *VEGF* expression in gliomas with different *IDH* genotypes from CGGA database **(A, B)**. CGGA, Chinese Glioma Genome Atlas; *IDH*, isocitrate dehydrogenase; *VEGF*, vascular endothelial growth factor.

The CGGA database analysis showed the expression level of *VEGF* was lower in *IDH^mut^* gliomas (**Figure 1**).

### Glioma Samples’ Demographics

A total of 56 patients were included. Immunohistochemistry revealed 17 patients with *IDH1^mut^* and 39 patients with *IDH1^wt^* gliomas (**Table 1**).

**Table 1:** Pathology and *IDH1* genotypes of glioma samples.

Grades	Total	*IDH1^mut^*	*IDH1^wt^*
G2	14	12	2
G3	3	2	1
G4	39	3	36
Total	56	17	39

G2, glioma grade II; G3, glioma grade III; G4, glioma grade IV; IDH, isocitrate dehydrogenase; IDH1^mut^, IDH1 mutant type; IDH1^wt^, IDH1 wildtype.

### Comparisons of Histogram Parameters of *K^trans^*, *V_e_* and *VEGF* Expression Between *IDH1^mut^* and *IDH1^wt^* Gliomas

**Figure 2:**
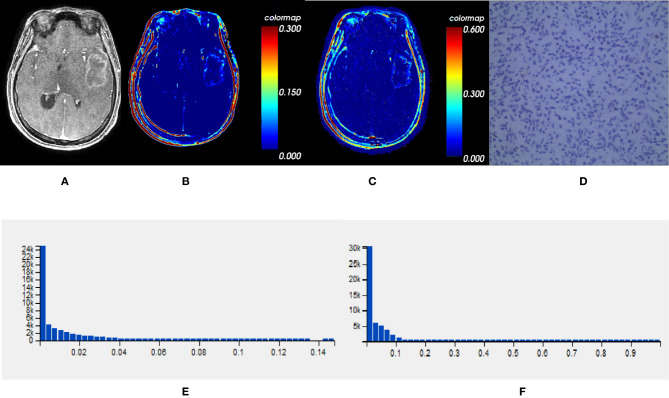
One *IDH1^mut^* glioblastoma in left temporal lobe; **(A)** T1-weighted contrast map, **(B)** *K^trans^* map, **(C)** *V_e_* map, **(D)** *VEGF* sample from lesion area, **(E)** Histogram distribution of *K^trans^*, **(F)** Histogram distribution of *V_e_*. The signal intensities of *K^trans^* and *V_e_* in the lesion area were lower than those of the wildtype counterpart. In *VEGF* sample from lesion area, the staining cells and intensities were significantly fewer and lower than the wildtype counterpart. *IDH*, isocitrate dehydrogenase; *IDH1^mut^*, *IDH1* mutant type; *K^trans^*, volume transfer constant; *V_e_*, volume of extravascular extracellular space per unit volume of tissue; *VEGF*, vascular endothelial growth factor.

**Figure 3:**
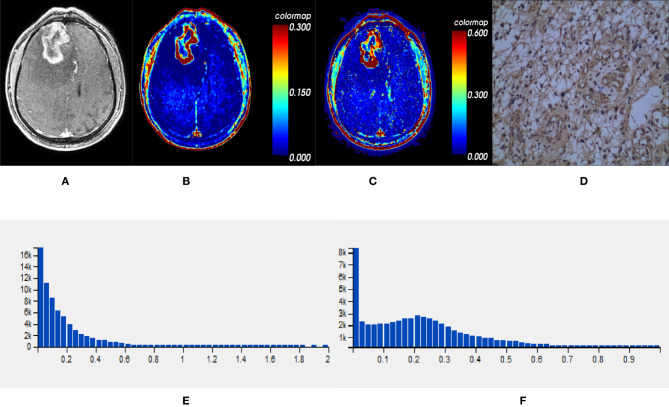
One *IDH1^wt^* glioblastoma in right frontal lobe; **(A)** T1-weighted contrast map, **(B)** *K^trans^* map, **(C)** *V_e_* map, **(D)** *VEGF* sample from lesion area, **(E)** Histogram distribution of *K^trans^*, **(F)** Histogram distribution of *V_e_*. The signal intensities of *K^trans^* and *V_e_* in the lesion area were higher than those of the mutant type counterpart. In *VEGF* sample from lesion area, the staining cells and intensities were significantly more and higher than those in the mutant type counterpart. *IDH*, isocitrate dehydrogenase; *IDH1^wt^*, *IDH1* wildtype; *K^trans^*, volume transfer constant; *V_e_*, volume of extravascular extracellular space per unit volume of tissue; *VEGF*, vascular endothelial growth factor.

Comparisons demonstrated that the mean, SD, 50th, 75th, 90th, and 95th percentile of *K^trans^*, *V_e_* and *VEGF* expression were significantly lower in *IDH1^mut^* than *IDH1^wt^* gliomas (*P* < 0.05, respectively) (**Table 2** and **Figures 2**, **3**).

**Table 2:** Histogram parameters and *VEGF* expression in *IDH1^mut^* and *IDH1^wt^*glioma samples.

Parameter		*IDH1^mut^*	*IDH1^wt^*	*P*
*K^trans^*(min^−1^)	Mean	0.024 ± 0.043	0.131 ± 0.144	<0.001
	SD	0.034 ± 0.052	0.154 ± 0.167	<0.001
	50th	0.013 ± 0.025	0.089 ± 0.103	<0.001
	75th	0.036 ± 0.065	0.174 ± 0.187	<0.001
	90th	0.065 ± 0.110	0.288 ± 0.297	<0.001
	95th	0.087 ± 0.143	0.393 ± 0.408	<0.001
*V_e_ (no units)*	Mean	0.077 ± 0.097	0.256 ± 0.185	<0.001
	SD	0.118 ± 0.101	0.212 ± 0.091	0.001
	50th	0.034 ± 0.050	0.175 ± 0.137	<0.001
	75th	0.096 ± 0.147	0.307 ± 0.194	<0.001
	90th	0.169 ± 0.224	0.451 ± 0.240	<0.001
	95th	0.225 ± 0.266	0.549 ± 0.251	<0.001
*VEGF* expression		1.290 ± 0.588	2.150 ± 0.670	<0.001

IDH, isocitrate dehydrogenase; IDH1^mut^, IDH1 mutant type; IDH1^wt^, IDH1 wildtype; K^trans^, volume transfer constant; SD, standard deviation; V_e_, volume of extravascular extracellular space per unit volume of tissue; VEGF, vascular endothelial growth factor. Data are presented as mean ± standard deviation.

### ROC Curve Analysis of Histogram Parameters of *K^trans^* and *V_e_* for Differentiating *IDH1^mut^* From *IDH1^wt^* Gliomas

**Figure 4:**
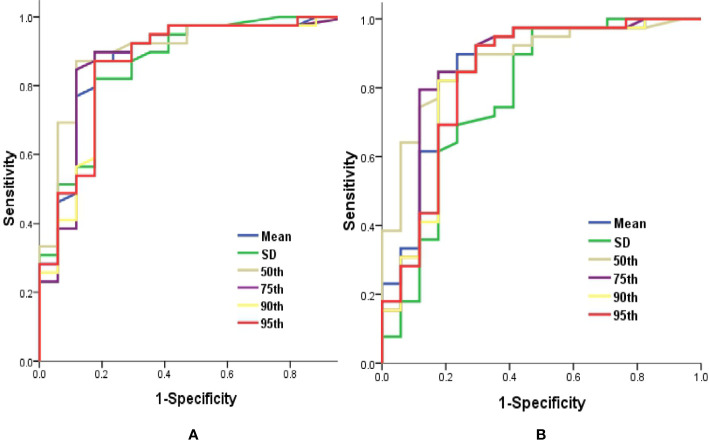
Graphs showed ROC curves of histogram parameters of *K^trans^* **(A)** and *V_e_* **(B)** for differentiating the *IDH1^mut^*from *IDH1^wt^* gliomas. *IDH*, isocitrate dehydrogenase; *IDH1^mut^*, *IDH1* mutant type; *IDH1^wt^*, *IDH1* wildtype; *K^trans^*, volume transfer constant; ROC, receiver operating characteristic; *V_e_*, volume of extravascular extracellular space per unit volume of tissue.

ROC curve analysis revealed that 50th percentile of *K^trans^* (0.019 min^−1^) and *V_e_* (0.039) provided the perfect combination of sensitivity (0.872, 0.846) and specificity (0.882, 0.824) in distinguishing *IDH1^mut^* with *IDH1^w^t* gliomas (*P* < 0.05, respectively). Area under the curve (AUC) for *K^trans^* and *V_e_* were 0.899 and 0.880 (**Table 3** and **Figure 4**).

**Table 3:** ROC curve analysis of histogram parameters**for differentiating *IDH1^mut^* from *IDH1^wt^* glioma samples.

Parameter		Cut-off	Sensitivity	Specificity	AUC	*P*
*K^trans^*(min^−1^)	Mean	0.035	0.872	0.824	0.882	<0.001
	SD	0.041	0.821	0.824	0.863	<0.001
	50th	0.019	0.872	0.882	0.899	<0.001
	75th	0.061	0.846	0.882	0.878	<0.001
	90th	0.076	0.872	0.824	0.862	<0.001
	95th	0.103	0.872	0.824	0.867	<0.001
*V_e_ (no units)*	Mean	0.095	0.897	0.765	0.858	<0.001
	SD	0.086	0.974	0.529	0.776	0.001
	50th	0.039	0.846	0.824	0.880	<0.001
	75th	0.152	0.795	0.882	0.863	<0.001
	90th	0.251	0.821	0.824	0.837	<0.001
	95th	0.242	0.923	0.706	0.833	<0.001

AUC, area under the curve; IDH, isocitrate dehydrogenase; IDH1^mut^, IDH1 mutant type; IDH1^wt^, IDH1 wildtype; K^trans^, volume transfer constant; ROC, receiver operating characteristic; SD, standard deviation; V_e_, volume of extravascular extracellular space per unit volume of tissue.

### Correlations Between Histogram Parameters of *K^trans^*, *V_e_* and *VEGF* Expression in Gliomas

Irrespective of *IDH1* mutation, histogram parameters of *K^trans^*and *V_e_* were positively correlated with *VEGF* expression in gliomas (*P* < 0.05, respectively) (**Table 4**).

**Table 4:** The correlations between histogram parameters and *VEGF* expression in glioma samples.

Parameter		All gliomas		*IDH1^mut^* gliomas		*IDH1^wt^*gliomas	
		*VEGF*	*P*	*VEGF*	*P*	*VEGF*	*P*
*K^trans^*(min^−1^)	Mean	r = 0.903	<0.001	r = 0.840	<0.001	r = 0.884	<0.001
	SD	r = 0.879	<0.001	r = 0.793	<0.001	r = 0.833	<0.001
	50th	r = 0.847	<0.001	r = 0.745	0.001	r = 0.813	<0.001
	75th	r = 0.893	<0.001	r = 0.834	<0.001	r = 0.878	<0.001
	90th	r = 0.909	<0.001	r = 0.840	<0.001	r = 0.898	<0.001
	95th	r = 0.911	<0.001	r = 0.847	<0.001	r = 0.898	<0.001
*V_e_ (no units)*	Mean	r = 0.874	<0.001	r = 0.800	<0.001	r = 0.844	<0.001
	SD	r = 0.821	<0.001	r = 0.789	<0.001	r = 0.776	<0.001
	50th	r = 0.794	<0.001	r = 0.701	0.002	r = 0.716	<0.001
	75th	r = 0.857	<0.001	r = 0.787	<0.001	r = 0.821	<0.001
	90th	r = 0.881	<0.001	r = 0.800	<0.001	r = 0.858	<0.001
	95th	r = 0.882	<0.001	r = 0.800	<0.001	r = 0.858	<0.001

IDH, isocitrate dehydrogenase; IDH1^mut^, IDH1 mutant type; IDH1^wt^, IDH1 wildtype; K^trans^, volume transfer constant; SD, standard deviation; V_e_, volume of extravascular extracellular space per unit volume of tissue; VEGF, vascular endothelial growth factor.

## Discussion

*IDH* mutation indicates a favorable clinical prognosis as compared to *IDH* without mutation in gliomas (12, 13). A previous study found that patients with *IDH1^wt^* anaplastic astrocytomas even exhibited worse prognosis than those with *IDH1^mut^* glioblastomas (14). Therefore, there are several differences between the two genotypes of glioma. Analysis of CGGA database and clinical practice in our hospital indicated that *VEGF* expression was significantly lower in *IDH1^mut^* as compared to the wildtype counterpart. Some studies demonstrated that the expression of *VEGF* was inhibited in the *IDH^mut^* gliomas (15, 16). These findings supported our results indirectly.

In recent years, several MRI techniques provide insight into exploring the link between glioma *IDH* mutation and microvascular characteristics. Conventional MRI showed the frequency and degree of enhancing were lower in gliomas with *IDH* mutation (17, 18). Moreover, radiomics served as predictive markers in assessing the *IDH* mutation in gliomas (19, 20). Dynamic susceptibility contrast-enhanced magnetic resonance imaging (DSC-MRI) and arterial spin labeling (ASL) revealed that blood flow or blood volume was significantly lower in *IDH^mut^* than *IDH^wt^* gliomas (3, 21–23). The current results implied a discriminant function for differentiating between *IDH1^mut^* and *IDH1^wt^* gliomas was constructed using DCE-MRI. Hilario et al. and Zhang et al. found that *K^trans^*was lower in gliomas with *IDH* mutation, which corroborates our results (24, 25).

Hitherto, there is no study focusing on the combined estimation of glioma *IDH1* mutation and *VEGF* expression with histogram analysis of DCE-MRI. In our study, we found that the signal intensities of lesions on *K^trans^* and *V*_e_ maps were lower in *IDH1^mut^* than *IDH1^wt^* gliomas. Simultaneously, the values of *K^trans^* and *V*_e_ were significantly higher in *IDH1^wt^* than *IDH1^mut^* gliomas. Our study also demonstrated that *K^trans^* and *V*_e_ were correlated with *VEGF* expression in gliomas. The conclusions clarifying *K^trans^* was correlated with *VEGF* expression in gliomas were consistent with our findings (26, 27). Pang et al. published that cerebral blood flow (CBF) derived from ASL also positively correlated significantly with *VEGF* expression in gliomas (28). Although both DCE-MRI and ASL can quantitatively assess tumor perfusion, they might be not completely comparable. The ASL primarily represents the degree of microvascular capacity, while microvascular immaturity and permeability are reflected by DCE-MRI. The pivotal features of *VEGF* are to enhance microvessel leakage and stimulate mitosis of endothelial cells to develop immature vascular infrastructure for supporting tumor cells (29, 30). However, the studies conducted by Awasthi et al. and Haris et al. did not demonstrate the positive correlation between *K^trans^* and *VEGF* expression in gliomas (31, 32). One reason might be that glioma samples were not exactly same. More glioblastomas were recruited in our study. The other might be that the methods used were different. The studies mentioned above chose partial areas of solid tumor as ROI, while entire tumor parenchyma was included in our study. More importantly, histogram analysis can assess the heterogeneity of tumors and improve the accuracy of diagnosis.

However, we have recognized a few limitations in the current study. First, the small sample size of glioma specimens restricts the accuracy of statistical analysis, and more samples are needed to verify the results in the future. Second, the *IDH2* mutation has not been considered due to the lack of genomic sequence analysis in our hospital. Owing to the low frequency of *IDH2* mutation in gliomas, the immunohistochemistry technique for detecting *IDH1* mutation could represent the maximum number of *IDH* mutation in gliomas (33). Nonetheless, gene sequencing should substantiate these results in following studies.

## Conclusions

*VEGF* expression is significantly lower in *IDH1^mut^* gliomas as compared to that in the wildtype counterpart, and it is non-invasively predictable with histogram analysis of DCE-MRI.

## Data Availability Statement

The raw data supporting the conclusions of this article will be made available by the authors without undue reservation.

## Ethics Statement

The studies involving human participants were reviewed and approved by the Ethics Committee of Affiliated Hospital of Nantong University. The patients/participants provided their written informed consent to participate in this study. Written informed consent was obtained from the individual(s) for the publication of any potentially identifiable images or data included in this article.

## Author Contributions

YH conceived and wrote the paper. YC processed the imaging. JW and JK analyzed the study data. DS and ZJ supervised and leaded the study. All authors contributed to the article and approved the submitted version.

## Funding

This work is supported by the China Postdoctoral Science Foundation (2014M551641) and Jiangsu Commission of Health (H2019089).

## Conflict of Interest

The authors declare that the research was conducted in the absence of any commercial or financial relationships that could be construed as a potential conflict of interest.

## Abbreviations

AUC, Area under the curve; ASL, Arterial spin labeling; CGGA, Chinese Glioma Genome Atlas; CBF, Cerebral blood flow; DCE-MRI, Dynamic contrast-enhanced magnetic resonance imaging; DSC-MRI, Dynamic susceptibility contrast-enhanced magnetic resonance imaging; *IDH*, Isocitrate dehydrogenase; *K^trans^*, Volume transfer constant; ROIs, Regions of interest; ROC, Receiver operating characteristic; *V_e_*, Volume of extravascular extracellular space per unit volume of tissue; *VEGF*, Vascular endothelial growth factor; WHO, World Health Organization.
